# Metformin Use and Metformin-associated Lactic Acidosis in Intensive Care Unit Patients with Diabetes

**DOI:** 10.7759/cureus.4739

**Published:** 2019-05-23

**Authors:** Venkat Rajasurya, Humayun Anjum, Salim Surani

**Affiliations:** 1 Pulmonary Critical Care, Decatur Memorial Hospital, Decatur, USA; 2 Pulmonary Critical Care, Corpus Christi Medical Center, Corpus Christi, USA; 3 Internal Medicine, Texas A&M Health Science Center, Temple, USA

**Keywords:** metformin associated lactic acidosis, acute kidney injury, diabetes, metformin, icu, sepsis, metformin toxicity, blood glucose, antihyperglycemic, insulin

## Abstract

Metformin is a very potent anti-diabetic drug that has become the drug of choice for the treatment of type 2 diabetes. In addition to its glucose-lowering properties, it also reduces all-cause mortality through its anti-inflammatory and cardioprotective effects. Although metformin-associated lactic acidosis (MALA) is a very rare event, the mortality associated with it is close to 50%. As it is excreted through the kidney, MALA is frequently seen in patients on metformin with risk factors for developing acute kidney injury. Metformin increases the plasma lactate level in a concentration-dependent manner by inhibiting mitochondrial respiration, usually in the presence of a secondary event that disrupts lactate production or clearance. The incidence of acute kidney injury is very high in critically ill patients contributed by circulatory defects as well as contrast-induced nephropathy, the incidence of which is also high in this subset of the population. Because of this potential risk, metformin is frequently discontinued in diabetic patients admitted to the intensive care unit. Blood glucose variability and hypoglycemia, however, are both related to poor intensive care unit (ICU) outcomes and in order to prevent this in diabetic patients admitted to ICU, oral hypoglycemic agents are frequently switched to intravenous or subcutaneous insulin regimens, which allows for closer monitoring and better blood glucose control.

## Introduction and background

Metformin and phenformin are derivatives of galegine, which was used in herbal medicine in medieval Europe [1]. The most significant benefit of metformin is reduction in all-cause mortality [2]. But, other benefits include relatively low cost, absence of weight gain, low level of side effects, and anti-inflammatory and antioxidant properties. Hence, it is recommended as the first line treatment [3]. Despite all the benefits, metformin is contraindicated in a certain subgroup of patients. It can cause lactic acidosis, which is rare, but can be potentially fatal [4]. Most commonly, it occurs in patients with a condition that impairs the lactate production or its clearance. Critically ill patients who are on metformin are at a higher risk for this complication as they are more prone to develop acute kidney injury (AKI), hepatic failure, respiratory failure, and circulatory shock in an intensive care unit (ICU) setting. With the increase in the percentage of elderly patients being managed in the ICU, this potential risk further increases. To avoid potential complications and to achieve a better blood glucose control, metformin is commonly switched to insulin in ICU patients.

## Review

Metformin metabolism and mechanism of action

Metformin is a unique anti-diabetic drug, as it does not cause hypoglycemia [2]. This is due to its characteristic pharmacokinetics. Following oral administration of metformin, a major proportion is absorbed and the rest is excreted in the stool. Additionally, almost all of the absorbed drug is eliminated unchanged via kidneys. The major site of action of metformin is the liver [5]. It not only reduces the glucose output by inhibiting the endogenous glucose production but also augments glucose uptake in the peripheral tissues due to improved insulin sensitivity [6].

Metformin and lactic acidosis

Lactic Acidosis

**Figure 1:**
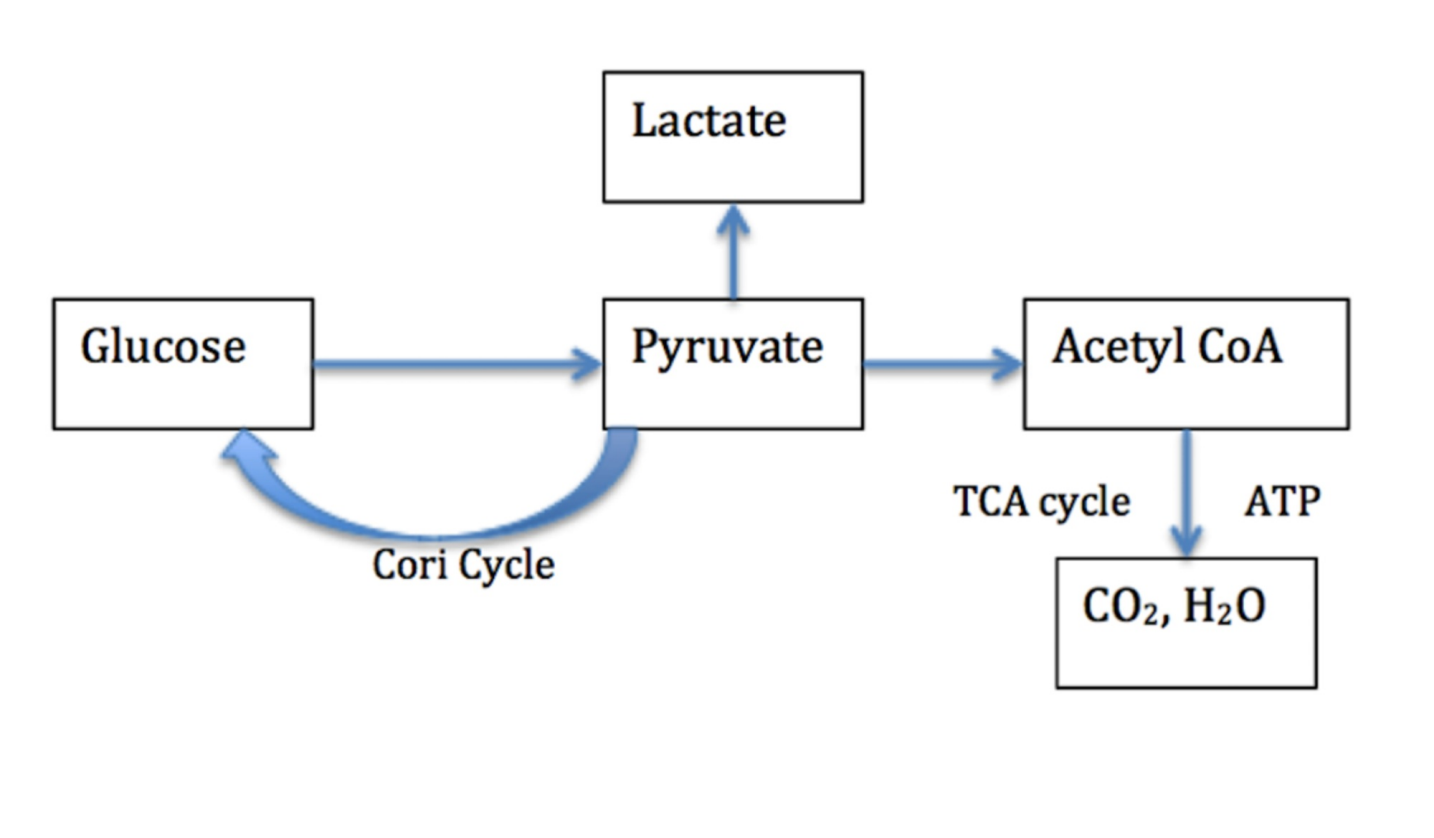
Biochemistry of lactate production Pyruvate is produced from glucose via glycolysis. (1) Under adequate oxygen availability, pyruvate is oxidized to CO2 and H2O in the tricarboxylic acid (TCA) cycle (2) Under anaerobic conditions, pyruvate is unable to enter mitochondria and is reduced to lactate. (3) Pyruvate can be converted to glucose in the liver and kidney by the Cori cycle. Lactate accumulation occurs under anaerobic conditions.

Lactate accumulates during hypoxia because of glycolysis and is produced by various organs including but not limited to the gut, liver and peripheral tissues [7]. Figure 1 clearly outlines the biochemistry of lactate production. When there is sufficient oxygen supply, glucose is metabolized to form water and carbon dioxide. This process results in the production of adenosine triphosphate (ATP), which is the energy carrier of the cell. Hypoxic conditions promote increased glycolysis to provide additional ATP. The excess pyruvate produced during this process is converted into lactate and subsequently released into the bloodstream where it accumulates over time [7]. 

Metformin-induced Lactic Acidosis

Metformin has several effects on pathways involving lactate. Metformin inhibits gluconeogenesis by blocking pyruvate carboxylase and this leads to lactate accumulation [8]. It also augments lactate production in insulin-dependent tissue, promotes glycolysis due to mitochondrial impairment and activates anaerobic metabolism of glucose in the intestine. Under normal circumstances, at therapeutic levels of metformin, this has no significant effect on the accumulation of lactate as it is converted back to glucose via the Cori cycle. If there is an accumulation of metformin due to either defective elimination or excess intake, there is a decrease in lactate uptake by the liver and lactic acidosis ensues. Not everyone with an isolated metformin overdose develops lactic acidosis or significant hyperlactatemia [9].

Metformin-associated Lactic Acidosis (MALA)

MALA remains an important adverse event due to its increased mortality. Table 1 highlights the three likely scenarios in which patients treated with metformin can develop lactic acidosis [10]. 

**Table 1:** Conditions that may increase the risk of lactic acidosis associated with metformin

Mechanism	Clinical Conditions
Promote the formation of lactate by the peripheral tissues because of hypoxia	Cardiac failure, Sepsis, Severe dehydration, Respiratory failure
Impair lactate metabolism through the pathway of gluconeogenesis	Primary or secondary hepatic failure
Dramatically increase levels of metformin	Renal impairment

Erythrocyte levels and an assay of blood levels of metformin can be helpful to estimate the risk and extent of metformin accumulation. This can be extremely complicated and perhaps not very reliable due to broad range of observed concentrations and continuous distribution of values [11]. Additionally, there is not much data regarding this as well. MALA without an associated condition is exceptional and thus is not necessarily due to metformin accumulation.

Risk Factors for Mala

It is the imbalance between the production of lactate and reduced clearance of lactate leads to MALA. MALA needs to be considered when the metformin level is more than 5 μg/mL (therapeutic range <2 μg/mL) [12]. The common risk factors for MALA are listed in Table 2 [10]. In general, the metformin plasma concentrations are two to four-fold higher in patients with type 2 diabetes and moderate to severe renal impairment when compared to healthy subjects [13]. Type 2 diabetes puts people at greater risk for hyperlactatemia [14]. Therefore, diabetic patients who are treated with metformin have a higher tendency for the development of lactic acidosis in response to a secondary event. The cases of MALA reported in the literature were commonly encountered in ICU settings where there is an increased risk for developing acute kidney injury (AKI).

**Table 2:** Clinical scenarios causing lactic acidosis in patients treated with metformin

Clinical Scenario	Features
Metformin-unrelated lactic acidosis	Metformin is not detectable in the blood
Metformin-induced lactic acidosis	Causal factors other than marked metformin accumulation are absent
Metformin-associated lactic acidosis	Metformin detected in blood and other disease conditions are present

Epidemiology of AKI in the ICU

Acute kidney injury is defined as the deterioration of renal function over hours, days to weeks. The mortality rate of AKI is 50%-80% in intensive care unit patients [15]. AKI is an independent risk factor for increased mortality and morbidity [15]. A prospective multicenter ICU study found that patients with sepsis and AKI had a higher mortality rate (74% vs 45%, p<0.001) than those without sepsis [16]. AKI may be community acquired or hospital acquired. Hospital-acquired AKI is associated with a worse prognosis and is often iatrogenic in nature [17]. Amongst various high-risk settings that put patients at an increased risk of AKI, infections, sepsis, shock, need for mechanical ventilation and surgery are a few worth mentioning. In 2012, the Kidney Disease Improving Global Outcomes (KDIGO) Clinical Practice Guideline for AKI consolidated the Risk Injury Failure Loss ESRD (RIFLE) criteria and Acute Kidney Injury Network (AKIN) criteria into the most recent definition and classification system for AKI as shown in Table 3 [18]. The current definition and classification of AKI rely upon functional criteria including changes in serum creatinine and urine output. More than half of the patients in the ICU develop stage 1 AKI during the ICU course. The exact incidence of developing stage 2 and 3 AKI is not well known but is believed to be far less. And lastly, the requirement for renal replacement therapy is about 10% [15]. Amongst the various risk factors, age, anemia, liver failure, chronic kidney disease, presence of heart failure and exposures to nephrotoxic agents are notable [19].

**Table 3:** Kidney Disease Improving Global Outcomes (KDIGO) acute kidney injury (AKI) classification

	Creatinine criteria	Urine output criteria
Risk or Stage 1	Creat >0.3 mg/dl <48 hr or Creat >150% and <200 < 7 d	U/O <0.5ml/kg/h for 6h
Injury or Stage 2	Creat >200% and <300% <7 d	U/O <0.5ml/kg/h for 12 h
Failure or Stage 3	Creat > 300%, or >4 mg/dl <7 d	U/O <0.3 ml/kg/h for 24 h or anuria for 12 h

Incidence of MALA

The estimated incidence of MALA is 0.03 to 0.06 per 1000 patient-years [20]. Thus, it is very rare. Literature search clearly shows that the incidence of MALA is very low and even the few cases that have been reported were associated with conditions that would predispose to lactic acidosis.

A comparative outcomes study (The COSMIC Approach Study) that looked at patients with type-2 diabetes on metformin vs. patients on non-metformin anti-hyperglycemic therapies for 1 year, found no cases of lactic acidosis. They also reported that the incidence of other serious adverse events were similar between the two groups [21]. Another meta-analysis that included 347 comparative trials and cohort studies compared patients treated with metformin with those not on metformin and concluded no significant difference in the plasma lactate levels and there were no cases of lactic acidosis [22]. In a U.K. nested case-control analysis of 50,048 patients, using a U.K-based General Practice Research Database it was revealed that there was no difference in the incidence of lactic acidosis between metformin and sulfonylurea users [23]. Of note, lactic acidosis reported in this study occurred in patients with preexisting conditions. These studies excluded the patients with renal failure (serum creatinine more than 1.4 mg/dl). Clinical trials and case-control studies generally don’t include patients at risk for MALA and hence they don’t reflect the actual prevalence in the community. It is also important to understand that the true incidence of MALA is difficult to predict as there are inconsistencies in reporting metformin levels, creatinine levels, lactate levels and associated conditions [24].

Mechanism of MALA

Lactate gets oxidized to carbon dioxide and water by mitochondria to generate ATP or gets converted back to glucose by gluconeogenesis in the liver and kidney [8]. The liver can clear lactate at a very high rate and this far exceeds lactate production [7]. Therefore, lactic acidosis is rare just from increased peripheral lactate production. But, in the presence of impaired hepatic metabolism, as in liver cirrhosis, sepsis or hypoperfusion this can lead to clinically significant lactic acidosis. There are mainly two types of lactic acidosis as mentioned by Cohen and Woods. Type A usually occurs in a setting of poor tissue perfusion or oxygenation of blood and type B occurs when there is increased lactate production or reduced clearance as listed in Table 4 [25].

**Table 4:** Lactic acidosis types

Type	Subtype	Conditions
Type A (Tissue hypoxia)	Systemic hypoperfusion	Shock (hypovolemic, septic, cardiogenic)
	Local hypoperfusion	Torsion/volvulus, arterial embolism
	Reduced arterial oxygen content	Hypoxemia, severe anemia, carbon monoxide toxicity
Type B (No tissue hypoxia)	B1- Underlying disease	Severe liver disease, malignancy, thiamine deficiency, renal failure
	B2- Drugs/ Toxins	Biguanides, alcohol, cyanide, acetaminophen, ethylene glycol, salicylates, isoniazid, zidovudine
	Type B3- Congenital metabolic defects	Mitochondrial disorders

**Figure 2:**
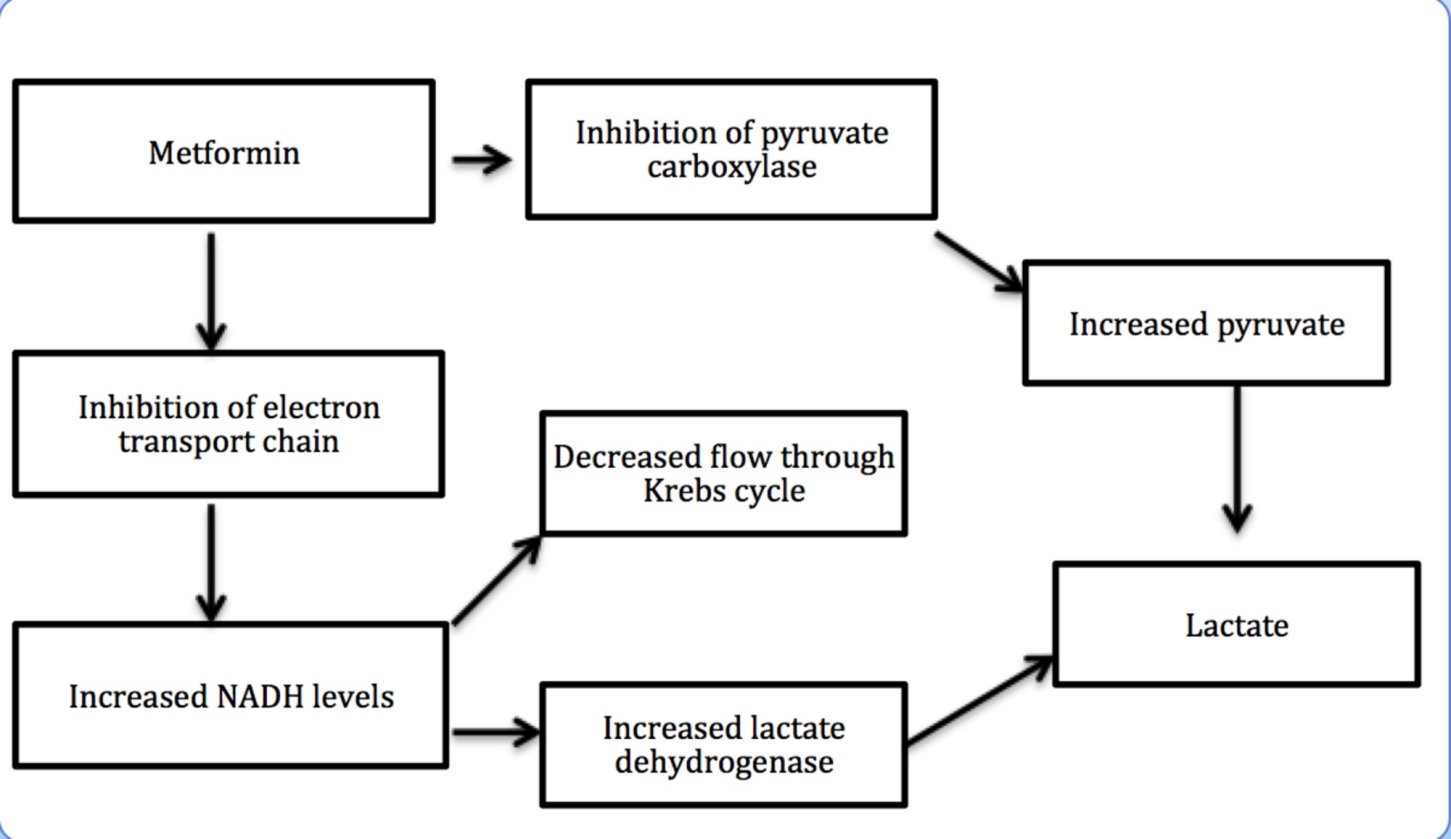
Mechanism of metformin-associated lactic acidosis (MALA) A. Metformin inhibits pyruvate carboxylase, which results in inhibition of hepatic gluconeogenesis and in turn leads to lactate accumulation. B. Metformin also inhibits complex I of the mitochondrial electron transport chain, which increases the nicotinamide adenine dinucleotide (NADH) level, that in turn reduces the flow through the Krebs cycle and also increases lactate dehydrogenase (LDH) activity resulting in lactate accumulation.

Metformin increases plasma lactate levels by inhibiting mitochondrial oxidative phosphorylation in tissues responsible for lactate removal. Metformin also inhibits hepatic gluconeogenesis, resulting in additional lactate production. This is explained in Figure 2 [8].

Metformin and cardiovascular diseases

In cardiovascular ICU we routinely see patients with diabetes who are on metformin. Metformin should be continued in patients with stable coronary artery disease, acute coronary syndrome, and CHF as it is associated with a better cardiovascular prognosis compared to other oral glucose-lowering agents. However, this does not apply to patients in acute circulatory failure due to the potential risk of tissue hypo-perfusion [26]. 

Metformin in patients with renal insufficiency

Metformin is excreted through the kidneys. Therefore, its dosage should be reduced in patients with renal insufficiency in proportion to the reduced eGFR. In chronic kidney disease, the elimination of metformin is prolonged and is inversely proportional to the creatinine clearance. This explains the risk of metformin accumulation in case of renal insufficiency [27].

Chronic kidney disease (CKD) is defined as the creatinine clearance of less than 60 mL/min. In patients with type-2 diabetes mellitus and CKD, initiation of metformin is a contraindication. Because of this contraindication, there is very limited data available regarding metformin use and the risk of lactic acidosis in patients with CKD. According to the Reduction of Atherothrombosis for Continued Health (REACH) Registry, which included a study sample of 19,691 type 2 diabetic patients with established atherothrombosis, metformin was prescribed in 1,572 patients with moderate renal failure (KDOQI stage 3) in contraindication to guidelines for its use. The results showed that when compared to other oral hypoglycemia agents, metformin use was associated with a significantly lower two-year mortality rate in patients with an eGFR of 30 to 60 mL/min/1.73 m2 and it was much greater than that observed in patients with normal renal function [28].

These results were further strengthened by similar findings observed in a large population-based observational study from the Swedish National Diabetes Register which looked at 51,675 patients with type 2 diabetes and different levels of renal function [29].

In this subgroup, particular attention should be paid to the renal function prior to initiation of metformin therapy and periodically thereafter using eGFR. Assess the benefits/risks of metformin use in patients with eGFR 30 to 45 mL/minute/1.73 m2; if used, dosage reduction is recommended [5].

A systematic review published in 2017, looked at patients with type 2 diabetes and moderate to severe CKD and analyzed the outcomes. All studies compared metformin-based treatments to non-metformin-based regimen. On meta-analysis, the relative chance of dying during follow-up was 22% lower for patients taking metformin than for those not taking metformin [30].

In April 2016, the FDA revised its safety warning regarding metformin use in patients with CKD, switching from a serum creatinine-based definition for renal impairment to more inclusive criteria based on estimated glomerular filtration rate (eGFR). With this change, an estimated one million additional patients with moderate CKD (eGFR 30-60 mL/min/1.73m2) became eligible for metformin use, though severe CKD (eGFR <30mL/min/1.73m2) still remains a contraindication. With the growing population in the moderate CKD group using the metformin, closer attention should be paid for any increase in adverse events [31].

Precautions for patients on metformin undergoing iodinated contrast imaging procedures in the ICU

AKI secondary to iodinated contrast is common in critically ill patients, with a reported incidence of 10% to 20%, with approximately 6% of those requiring renal replacement therapy [32]. The overall incidence today is decreasing because of various reasons. There has been more awareness, better preventive strategies, and availability of less toxic agents. Typically, AKI secondary to iodinated contrast is seen within three days and peaks in 5 days [18]. A small study evaluated 75 ICU patients with a normal baseline serum creatinine who were exposed to CT scans with an intravenous low osmolar contrast agent. The study found an increase in serum creatinine of more than 25% in 18% of the patients. On the other hand, patients who underwent CT scans and did not receive any contrast medium had no change in the serum creatinine [33]. 

According to the manufacturer, it is recommended to temporarily discontinue metformin at the time of or before iodinated contrast imaging procedures in patients with an eGFR 30 to 60 mL/minute/1.73 m2. This also applies to patients with a history of hepatic disease, alcoholism and heart failure. Patients who will receive intra-arterial iodinated contrast fall in this category as well. Reevaluation of eGFR 48 hours after imaging procedure is recommended and metformin can be restarted if the renal function is stable. The data regarding the use of iodinated contrast in patients who are on metformin is very scarce. A U.K. based study done in 1998 looked at 33 hospitalized patients who were on metformin and received iodinated contrast. Of these patients, 29 had a normal serum creatinine prior to the procedure and none of them had a rise following the procedure. The remaining four patients who had an abnormal serum creatinine prior to the procedure demonstrated a considerable deterioration and subsequently died. Two of these deaths were from unrelated causes and two were due to the development of acute renal failure [34].

On the other hand, the American College of Radiology (ACR) guidelines recommend that metformin may be used prior to or following administration of iodinated contrast media in patients who have no evidence of AKI and with an eGFR ≥30 mL/minute/1.73 m2. They do suggest temporary discontinuation of metformin in patients with known AKI or severe chronic kidney disease ([stage IV or V [ie, eGFR <30 mL/minute/1.73 m2]) or those who are undergoing arterial catheter studies (ACR 2017) [35].

Metformin interaction with ICU drugs

Metformin does not have any clinically important interactions with other drugs. However, there are certain medications which are cationic agents and may compete with metformin for elimination as they are eliminated by renal tubular secretion. Some of them are amiloride, digoxin, morphine, procainamide, quinidine, quinine, ranitidine, triamterene, trimethoprim, and vancomycin. Drugs such as cimetidine, furosemide, or nifedipine may increase the concentration of metformin if taken concomitantly. So, it is important to closely monitor patients who are on metformin in association with these agents for any such potential toxicity [36].

Blood glucose in ICU

Hyperglycemia, which is defined as blood glucose concentration [BG] >110mg/dL, is very common in the ICU and occurs in more than 80% of critically ill patients [37]. Hospitalization of patients with diabetes frequently leads to hyperglycemia or hypoglycemia by interrupting the outpatient balance of medications and diet [38]. Stress hyperglycemia occurs in hospitalized patients without evidence of pre-existing diabetes and it results from the acute metabolic and hormonal changes in response to stress and injury. There are several factors that affect blood glucose concentration in ICU patients. These include history of diabetes; insulin resistance; severity of the acute illness; enteral or parenteral glucose administration; use of steroids etc [39]. It has been well established that both variability in blood glucose levels and hypoglycemia are associated with increased mortality [40]. In light of this, the current Surviving Sepsis Campaign recommends that all patients with severe sepsis who have blood glucose levels that exceed 180mg/dL to be started on intravenous insulin therapy. The goal is usually to maintain blood glucose between approximately 144 to 180 mg/dL [41].

Stress hyperglycemia causes increased mortality in the setting of acute myocardial infarction. Similarly, in patients who are undergoing coronary artery bypass surgery, it has been shown that perioperative glycemic control correlates with postoperative risk of nosocomial infection [42]. The rate of post-op wound infection decreased with maintaining a goal blood glucose level of less than 200 mg/dl in diabetic patients undergoing cardiac surgery [43].

Management of hyperglycemia in a critical care setting

The overall goal is to minimize blood glucose variability without causing hypoglycemia. Current recommendations suggest keeping the blood sugar below 180 mg/dL and when insulin is needed to do so, to target a blood sugar of 144-180 mg/dL [44]. In the ICU patients with diabetes, who are hyperglycemic, it is recommended to start an intravenous insulin infusion [45]. This can be demanding but it helps minimize variability in blood glucose levels, which is a common occurrence in critically ill patients. If this regimen cannot be initiated, then alternatively a long-acting subcutaneous insulin with additional short-acting insulin can be utilized [38]. Insulin pumps should be discontinued in ICU due to the risk of hypoglycemia [38].

Preadmission metformin use and ICU mortality

It is very well accepted that metformin is a great hypoglycemic agent when it comes to glycemic control. But beyond that, there is limited data regarding its benefits. The most notable benefits are its proposed anti-inflammatory effects. There are numerous proposed theories that exist regarding the possible mechanism of this effect. In a review submitted by Saisho et al., it is suggested that there is improvement in chronic inflammation by improvement of metabolic parameters. It is also proposed that metformin has direct anti-inflammatory effects and the decreased incidence of hypoglycemic events leads to a decreased sympatho-adrenergic response [46].

Christiansen and colleagues conducted a population-based study and identified the cohort by using the criteria of filled metformin prescriptions 90 days prior to the admission to ICU. It was noted that the thirty-day mortality was 17.6% in patients using metformin alone, 17.9% in patients using metformin with other oral hypoglycemic agents and 25.0% in metformin non-users. This was true for all subgroups, including those in the medical and surgical ICUs. But, interestingly it was most significant in elderly patients and in patients with well-controlled diabetes. They concluded that metformin use prior to the admission was associated with a reduction in 30-day mortality [47].

Similarly, another cohort study done in the US on elective cardiac surgery patients found that patients on metformin had fewer postoperative complications and lower in-hospital mortality (0.7% versus 1.4%) [48].

Although there are several proposed mechanisms linking preadmission metformin use and improved critical care outcomes secondary to its anti-inflammatory and anti-thrombotic effects, further studies are required to establish clinical outcomes.

## Conclusions

In all current clinical guidelines, metformin is recommended as the first line oral hypoglycemic agent for the management of type 2 diabetes mellitus. But the main limiting factor has been the fear of MALA, especially in patients with CKD. MALA has a high mortality rate and therefore remains a concern, despite the fact that it is extremely uncommon. It is important to realize that MALA happens under conditions that promote hypoperfusion or hypoxia such as sepsis, dehydration or worsening renal, or cardiac failure; in the presence of these conditions, metformin should be promptly discontinued. Further studies are needed to assess whether routine discontinuation of metformin upon ICU admission should be considered.
